# FOXO in *Lymnaea*: Its Probable Involvement in Memory Consolidation

**DOI:** 10.3390/biology12091201

**Published:** 2023-09-01

**Authors:** Junko Nakai, Kengo Namiki, Kanta Fujimoto, Dai Hatakeyama, Etsuro Ito

**Affiliations:** 1 Department Biology, Waseda University, Tokyo 162-8480, Japan; kbc-jun5211@ruri.waseda.jp (J.N.); k-namiki38295k2r@ruri.waseda.jp (K.N.); k-fujimoto@akane.waseda.jp (K.F.); 2 Laboratory of Biochemistry, Faculty of Pharmaceutical Sciences, Tokushima Bunri University, Tokushima 770-8514, Japan; daihatake926@ph.bunri-u.ac.jp

**Keywords:** conditioned taste aversion, food deprivation, FOXO, insulin, *Lymnaea*

## Abstract

**Simple Summary:**

Insulin in the central nervous system (CNS) affects learning and memory, but the involvement of forkhead box O (FOXO), a transcription factor belonging to the insulin signaling cascade, is unclear. We identified FOXO in the pond snail *Lymnaea stagnalis* and examined its subcellular localization in CNS neurons. In the CNS, FOXO was observed in neuronal nuclei in food-deprived snails and in the neuronal cytoplasm of food-satiated snails. FOXO translocated to the nuclei in food-satiated snails when they were subjected to conditioned taste aversion (CTA) learning. Although insulin administration to CNSs isolated from food-satiated snails was expected to induce the translocation of FOXO into nuclei, FOXO remained in the cytoplasm. In addition, insulin administered to the CNSs upregulated only the expression of FOXO mRNA and not its related molecules. Together, these findings suggest that food deprivation prepares FOXO to function in neuronal nuclei and enhances CTA learning in snails. Insulin administration, however, may stimulate other pathways that are not downstream of the FOXO response cascade.

**Abstract:**

Food deprivation activates forkhead box O (FOXO), a transcription factor downstream of insulin receptors. In the pond snail *Lymnaea stagnalis*, insulin signaling and food deprivation improve memory consolidation following conditioned taste aversion (CTA) learning. We investigated the subcellular localization of FOXO in *Lymnaea* and changes in its expression levels following food deprivation, CTA learning, and insulin administration. Immunohistochemistry revealed that *Lymnaea* FOXO (LymFOXO) was located in the central nervous system (CNS) neuronal cytoplasm in food-satiated snails but was mainly in neuronal nuclei in food-deprived snails. Following CTA acquisition, LymFOXO translocated to the nuclei in food-satiated snails and remained in the nuclei in food-deprived snails. Contrary to our expectations, insulin administered to the CNS did not induce LymFOXO translocation into the nuclei in food-satiated snails. Real-time PCR was used to quantify LymFOXO mRNA levels, its target genes, and insulin signaling pathway genes and revealed that LymFOXO mRNA was upregulated in food-deprived snails compared to food-satiated snails. Insulin applied to isolated CNSs from food-satiated snails increased LymFOXO compared to vehicle-treated samples. Food deprivation prepares FOXO to function in the nucleus and enhances CTA learning in snails. Insulin application did not directly affect LymFOXO protein localization. Thus, insulin administration may stimulate pathways other than the LymFOXO cascade.

## 1. Introduction

In both vertebrates and invertebrates, learning and memory mechanisms are affected by insulin or insulin-like peptides in the central nervous system (CNS) [1,2,3,4,5]. For example, in the nematode *C. elegans*, which is frequently used as a model animal to clarify the molecular mechanisms of learning and memory, the main components of the insulin signaling pathway include INS-1 (insulin-like peptide), DAF-2 (insulin receptor), AGE-1 (phosphoinositide 3-kinase (PI3K)), DAF-16 (forkhead box protein O (FOXO)), DAF-18 (phosphatase and tensin homolog deleted on chromosome 10 (PTEN)), and AKT-1 (serine/threonine kinase) [6,7,8,9,10,11,12]. Investigations of the detailed functions of the transcription factor FOXO [13], however, have not clarified its involvement in memory consolidation [14]. Although FOXO acts downstream in the insulin signaling pathway, it is activated by food deprivation [15,16] and is located outside of the nucleus even after its phosphorylation [17]. Thus, the function of FOXO remains a mystery, and it probably has two different roles.

Insulin-like peptides (i.e., molluscan insulin-related peptides (MIPs)) also contribute to learning and memory formation in the pond snail *Lymnaea stagnalis* [18]. *Lymnaea* are capable of learning conditioned taste aversion (CTA) and consolidating it into long-term memory [19]. To produce CTA in *Lymnaea*, an appetitive stimulus (e.g., sucrose) is used as the conditioned stimulus (CS). Application of the CS to the lips increases the feeding response (i.e., the number of bites) in snails. An aversive stimulus (e.g., KCl) is used as the unconditioned stimulus (US). Application of the US to the snails inhibits feeding behavior. In the CTA training procedure, the CS is paired with the US. After repeated temporally contingent presentations of the CS and US, the CS no longer elicits a feeding response. During the consolidation of CTA into long-term memory, MIP gene expression is upregulated [20] and a synapse involved in CTA is strengthened by the application of MIPs or mammalian insulin [21]. Insulin also influences the behavioral change induced by CTA in *Lymnaea* [22]. Therefore, *Lymnaea* are ideal for studying the function of FOXO in the relationship between memory consolidation and the insulin pathway.

In the present study, we determined the mRNA sequence of *Lymnaea* FOXO (LymFOXO) and deduced its amino acid sequence and the existence of its mRNA and protein in the central nervous system (CNS). We then examined changes in the subcellular localization and expression levels of LymFOXO caused by food deprivation, CTA learning, and insulin administration in *Lymnaea*. Our findings suggest that LymFOXO enhances memory consolidation.

## 2. Materials and Methods

### 2.1. Animal Rearing and Food Deprivation

Specimens of *Lymnaea stagnalis* with 20 to 25 mm shell lengths obtained from our snail-rearing facility (original stocks from Vrije Universiteit Amsterdam) were used in the present study. All snails were maintained in dechlorinated tap water (i.e., pond water) under a 12/12 h light/dark cycle at 20–23 °C and fed ad libitum on turnip leaves (*Brassica rapa* var. *peruviridis*, known as Komatsuna in Japanese). Snails fed ad libitum were called Day –1 snails, whereas 1-day food-deprived snails were called Day 1 snails.

### 2.2. Identification of LymFOXO

Based on the transcriptome shotgun assembly (TSA) data for the Lymnaea CNS obtained in previous studies [23,24], we searched for Lymnaea mRNA sequences homologous to the Aplysia californica FOXO mRNA (accession number: XM_005112403) using NCBI-BLAST (https://blast.ncbi.nlm.nih.gov/Blast.cgi accessed on 4 August 2023). Four sequences (FX199776.1, FX255204.1, FX227609.1, and FX217506.1) were selected. The whole CNS was collected, and total RNA was extracted using ISOGEN II (311-07361; Nippon Gene, Tokyo, Japan) according to the manufacturer’s instructions. cDNA was synthesized using ReverTra Ace qPCR RT Master Mix with gDNA Remover (Toyobo, Osaka, Japan). PCR was performed to examine whether these 4 mRNA fragments were connected using FOXO (i) and FOXO (ii) primers (Table 1) and the PCR enzyme TaKaRa Ex Taq (Takara Bio, Shiga, Japan). The 4 fragments were confirmed to connect into one, but because there was no termination codon for the open reading frame (ORF), we determined that the 3′ terminal was missing. Thus, 3′ RACE experiments were performed. Reverse transcription was performed using Lymnaea total RNA, a 3′ RACE adaptor (Table 1), and the 3′-Full RACE Core Set (Takara Bio). The thermal cycle was set at 30 °C for 10 min, 50 °C for 60 min, and 80 °C for 2 min. The first PCR was performed using our prepared reverse-transcription reaction solution with a combination of a 3′ RACE FOXO-specific outer primer and a 3′ RACE outer primer (Table 1), and the second PCR was performed using the first PCR reaction solution with a combination of a 3′ RACE FOXO-specific inner primer and a 3′ RACE inner primer (Table 1). Both PCRs were performed using Ex Taq (Takara Bio) with the following protocol: 30 cycles of denaturation at 94 °C for 30 s, annealing at 55 °C for 30 s, and extension at 72 °C for 3 min.

PCR products were confirmed with agarose gel electrophoresis and purified with NucleoSpin Gel and PCR Clean-up (Macherey-Nagel, Dueren, Germany). A sequencing analysis was contracted to Eurofins (Tokyo, Japan). The ORF was determined using ApE (A plasmid Editor, https://jorgensen.biology.utah.edu/wayned/ape/ accessed on 4 August 2023). Neighbor-joining trees of FOXO-like proteins identified from different organisms were generated using MEGA 11 software (https://www.megasoftware.net/ accessed on 4 August 2023). The amino acid sequences used for the phylogenetic tree are listed in Table 2.

In addition, based on the TSA data for the *Lymnaea* CNS, we searched for *Lymnaea* mRNA sequences homologous to other molluscan G6Pase, PEPCK, catalase, PI3KCA, AKT, and mTOR mRNA using NCBI-BLAST. We called them LymG6Pase, LymPEPCK, LymCatalase, LymPI3K, LymAKT, and LymmTOR. Here, we noted that the PI3CA gene corresponded to p110α for PI3K. The accession number of each gene, the accession number of *Lymnaea*, and the amino acid identity are shown in Table 3.

### 2.3. Real-Time PCR

The real-time PCR protocol was performed as described previously with modifications [24,25]. CNSs were dissected from the snails and stored at −80 °C. In experiments to identify the ganglia expressing the target mRNAs, the samples were separated by ganglion type, and 3 individual pairs of ganglia were collected together as 1 sample, whereas whole CNSs were collected in other experiments. Total RNA was extracted using ISOGEN II (311-07361; Nippon Gene, Tokyo, Japan) according to the manufacturer’s instructions. cDNA was synthesized using ReverTra Ace qPCR RT Master Mix with gDNA Remover (Toyobo). THUNDERBIRD Next SYBR qPCR Mix (Toyobo) was used to perform real-time PCR (StepOnePlus Real-Time PCR System; Applied Biosystems, Waltham, MA, USA). Relative mRNA levels were quantified using the comparative CT method. The mRNA levels were normalized to the average value of 18S ribosomal RNA and β-tubulin RNA or that of β-actin and β-tubulin RNAs. These reference genes were stable in our experiments. The primer sequences are shown in Table 4. The efficiency values for the real-time PCR primers ranged from 90 to 110%. The PCR conditions were as follows: 1 cycle at 95 °C for 30 s, followed by 40 cycles of denaturation at 95 °C for 5 s, and annealing at 60 °C for 10 s. A melting curve analysis was performed from 60 to 95 °C with a heating rate of 0.3 °C/s.

### 2.4. Immunohistochemistry

The immunohistochemistry protocol was performed as described previously with modifications [26,27]. Frozen sections were prepared as follows: The CNS was isolated from each anesthetized *Lymnaea* in chilled saline (50 mM NaCl, 1.6 mM KCl, 2 mM MgCl_2_, 3.5 mM CaCl_2_, 10 mM HEPES, pH 7.9), fixed in 4% paraformaldehyde in phosphate-buffered saline (PBS) for 1 h at room temperature, and then washed with PBS containing 30% sucrose. After embedding the fixed CNS in an O.C.T. compound (Sakura Finetek, Tokyo, Japan), serial 10 µm thick frozen sections were cut horizontally on a cryostat (CM3000; Leica, Nussloch, Germany) and placed on MAS-coated glass slides (Matsunami glass, Osaka, Japan). An anti-FOXO3 rabbit polyclonal antibody (Abcepta, San Diego, CA, USA; 1:50 dilution) was used as the primary antibody. The efficacy of this primary antibody against LymFOXO was tested as follows: Western blotting was performed to confirm the specificity of the primary antibody toward LymFOXO using proteins extracted from the *Lymnaea* CNS. The results showed a band around 70.7 kDa, which is the molecular mass of LymFOXO. When absorption tests were carried out using the blocking peptide for the primary antibody (Abcepta), the band around 70.7 kDa disappeared. Then, an Alexa Fluor 488-labeled goat anti-rabbit IgG H&L antibody (Abcam, Cambridge, MA, USA; 1:1000 dilution) was used as the secondary antibody. Frozen sections were counterstained with 4′-6 diamino-2 phenylindole (DAPI; Roche, Basel, Switzerland).

### 2.5. Conditioned Taste Aversion Paradigm

The experimental protocol was performed as described previously with modifications [19]. The snails used for the behavioral experiments were Day −1 and Day 1 snails. The pretest, training, and post-test were performed in a polystyrene petri dish (diameter: 35 mm). All snails were acclimatized in distilled water (DW) for 30 min and given a pretest. In the pretest, 2 mL of a 10 mM sucrose solution was applied to the lips of the snail for 15 s as a conditioned stimulus (CS), and the number of feeding responses (i.e., rasping movements of the buccal mass) in DW was counted for 1 min. CTA learning in *Lymnaea* was induced by pairing a 10 mM sucrose solution as the CS and a 10 mM KCl solution as an unconditioned stimulus (US). This is called forward conditioning. The durations of both the CS and US were 15 s, with a 15 s interstimulus interval between the onset of the CS and the onset of the US. Snails received 10 paired CS-US trials with 10 min intertrial intervals. To validate the associative learning, backward-trained (i.e., US-CS) and naive control groups (i.e., DW-DW) were included. In the post-test session, snails were again challenged with the CS, and the number of bites was recorded for 1 min after a 15 s application of the CS. All behavioral experiments were performed in the morning, and the post-tests were performed with investigators blind to the group condition.

### 2.6. Drug Administration

The drug administration protocols were performed as described previously with modifications [21]. Briefly, 100 nM bovine insulin (Sigma-Aldrich, St. Louis, MO, USA) or 120 nM HCl (vehicle for bovine insulin) in *Lymnaea* saline were incubated with the isolated CNS for 1 h at room temperature. We could not use molluscan-related insulin peptides and thus used mammalian insulin instead. Previous experiments demonstrated that mammalian insulin is an adequate alternative to MIPs in the *Lymnaea* CNS [21] because the binding site of the *Lymnaea* MIP receptor is well conserved across phyla. For example, in a comparison with mammalian insulin receptors (accession numbers: CAA59353 for *Lymnaea*, AAA59174 for humans, and AAA39318 for mice) [28], the homology was 34% for the whole amino acid sequences, 56% for ligand-binding domain 1 (L1 domain), and 33% for ligand-binding domain 2 (L2 domain) between *Lymnaea*, humans, and mice. Previous studies using another mollusk, *Aplysia*, also demonstrated that the application of bovine insulin activated the bag cell-neuron insulin receptor by stimulating its autophosphorylation on tyrosine residues [28] and evoked egg-laying hormone secretion [29]. Furthermore, according to a previous study [21], the results of electrophysiological experiments in which bovine insulin or MIPs were administered showed that both caused long-term enhancement of the specific synaptic strength in the *Lymnaea* CNS. Totani’s research showed that the glucose content of the hemolymph was decreased by the administration of bovine insulin or MIPs [22]. More specifically, when bovine insulin was administered to the isolated *Lymnaea* CNS, our results clearly showed an increase in the phosphorylation of AKT and RICTOR in the PI3K/AKT/mTOR signaling cascade (submitted). Taken together, we believe that bovine insulin can be used in *Lymnaea* as well as MIPs.

### 2.7. Statistics

The data are expressed as means ± SEMs, with *p* < 0.05 considered statistically significant. Data from multiple groups were analyzed using one-way or two-way ANOVAs followed by a post hoc Holm’s test, and data from 2 groups were analyzed using Welch’s *t*-test. The computer software used was R (version 4.2.1; https://www.r-project.org/ accessed on 4 August 2023).

## 3. Results

### 3.1. Deduced Amino Acid Sequence of LymFOXO

**Figure 1:**
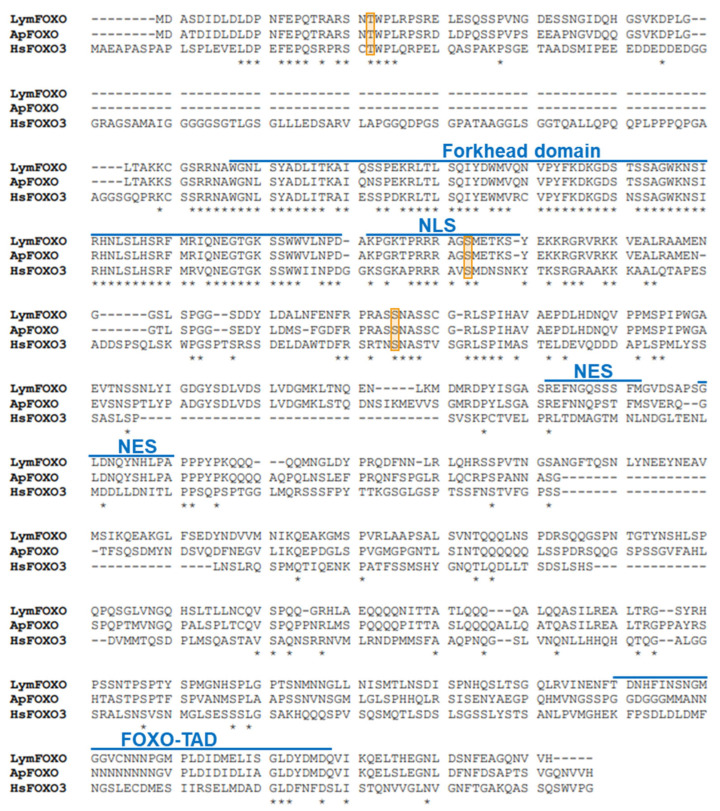
Comparison of deduced amino acids of LymFOXO with ApFOXO and HsFOXO3. NLS indicates a nuclear localization signal; NES indicates a nuclear export signal; FOXO-TAD indicates a transactivation domain of the FOXO protein family. Orange frames indicate the phosphorylation sites (Thr32, Ser253, and Ser315) of HsFOXO3 by AKT. * indicates conserved amino acids.

A putative FOXO in *Lymnaea*, LymFOXO, was searched for using *Aplysia californica* FOXO (ApFOXO) (XM_005112403) as a query sequence. The BLAST search resulted in a hit in the *Lymnaea stagnalis* mRNA TSA database (accession numbers: FX199776.1, FX255204.1, FX227609.1, and FX217506.1). The 3′ terminal was missing in these TSA sequences. Thus, 3′ RACE experiments were performed, and the whole open reading frame (ORF) was obtained using the ApE software. We registered LymFOXO in GenBank under accession number LC773945.1. The deduced amino acid sequence was aligned with ApFOXO and *Homo sapiens* FOXO3 (HsFOXO3) (Figure 1). We used HsFOXO3, corresponding to LymFOXO, because *C. elegans* and *Drosophila melanogaster* FOXO are orthologues of HsFOXO3 [13], and HsFOXO3 was hit by a BLAST search for human genes with high homology to LymFOXO. LymFOXO comprises 642 amino acids (MM. 70.7 kDa). LymFOXO and ApFOXO display 65.8% amino acid identity; LymFOXO and HsFOXO3 display 25.2% amino acid identity. The domain sequence predictions suggest that LymFOXO has a forkhead domain and conserves the amino acids phosphorylated by AKT across phyla [17].

**Figure 2:**
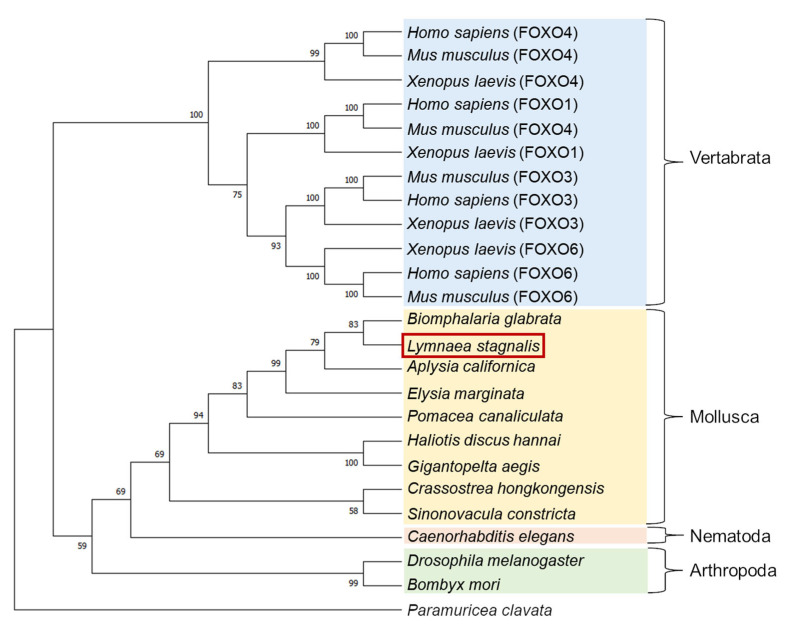
A molecular phylogenetic tree of 25 FOXO-like proteins using the neighbor-joining method. The bootstrap value of each branch was calculated by testing the phylogenetic tree 1000 times and is expressed as a percentage. The red square indicates the animal used in the present study.

A molecular phylogenetic tree of FOXO-like proteins deduced from various animals was generated using the neighbor-joining method [30] (Figure 2). LymFOXO was most closely related to the FOXO of another freshwater snail, *Biomphalaria glabrata*, and together with other molluscan FOXOs, they were clustered into one family and called the Mollusca group. This cluster forms the Invertebrata group with Nematoda and Arthropoda and differs from the Vertebrata group. Some kinds of FOXO have been identified in vertebrates. To our best knowledge, however, only one kind of FOXO can be found in invertebrates.

### 3.2. Confirmation of the Presence of LymFOXO mRNA in the Lymnaea CNS

**Figure 3:**
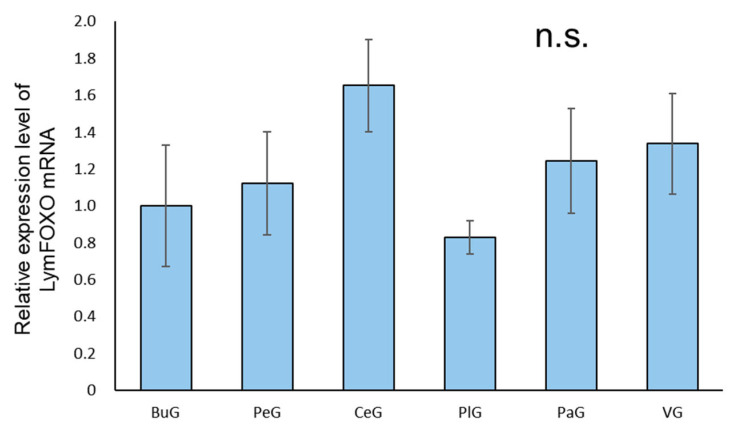
Relative amounts of LymFOXO mRNA in the ganglia of CNSs obtained from food-satiated Day−1 snails using real-time PCR. The mRNA expression levels were obtained after normalizing to 18S rRNA and β-tubulin, and the data are expressed relative to the expression in the buccal ganglia, which was set as 1 for easy comparison. BuG: buccal ganglion, PeG: pedal ganglion, CeG: cerebral ganglion, PlG: pleural ganglion; PaG: parietal ganglion, VG, visceral ganglion. *p* = 0.57. N = 3 groups each. One group consisted of 3 pairs of ganglia. n.s.—not significant.

A real-time PCR quantification of the mRNA extracted from each ganglion of food-satiated (Day−1) snails showed that LymFOXO was ubiquitously expressed in all the ganglia, and no significant difference was detected among the ganglia (*p* = 0.57, N = 3 groups each) (Figure 3).

### 3.3. Confirmation of the Presence of LymFOXO Protein in the Lymnaea CNS

To detect LymFOXO protein, we used an anti-FOXO3 rabbit polyclonal antibody, which was produced against amino acids 224–253 of HsFOXO3 as an epitope, and this epitope site is almost identical to amino acids 129–168 of LymFOXO (HsFOXO3: NLSLHSRFMRVQNEGTGKSSWWIINPDGGKSGKAPRRRAVS and LymFOXO: NLSLHSRFMRIQNEGTGKSSWWVLNPDAKPGKTPRRRAGS).

**Figure 4:**
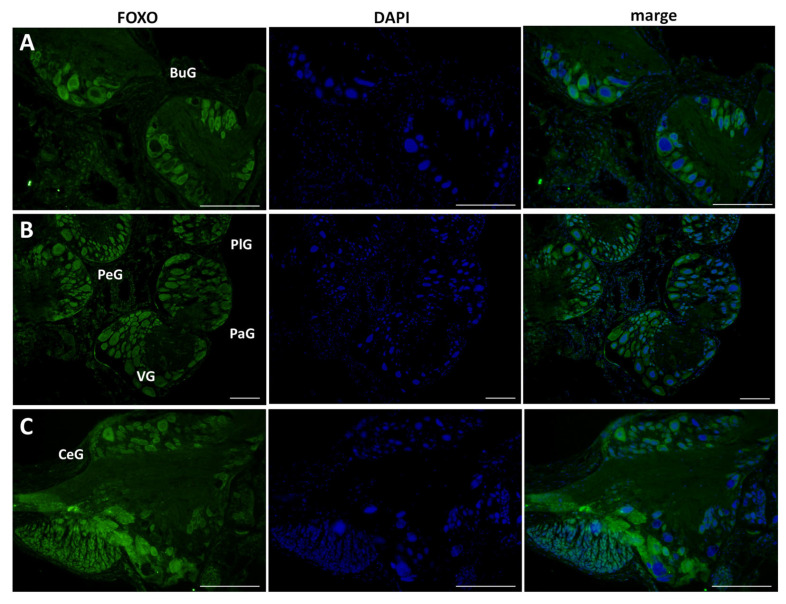
Confirmation of LymFOXO protein in CNS sections obtained from food-satiated Day −1 snails. (**A**) Buccal ganglion (BuG). (**B**) Pedal ganglion (PeG), pleural ganglion (PlG), parietal ganglion (PaG), and visceral ganglion (VG). (**C**) Cerebral ganglion (CeG). Green indicates the immunoreactivity of LymFOXO, and blue indicates DAPI. Scale bars: 100 µm.

This antibody was applied to 10 μm sections of CNS isolated from food-satiated (Day −1) snails, and immunohistochemistry was performed (Figure 4). LymFOXO protein was ubiquitously expressed in all the ganglia. Overall, LymFOXO was localized outside the nucleus, i.e., in the cytoplasm. In particular, the B4 cells in the buccal ganglia and the cerebral giant cells (CGCs) in the cerebral ganglia showed the presence of LymFOXO protein. No signal was observed in the negative control experiments in which only the secondary antibody was applied without the primary antibody.

### 3.4. Behavioral Changes Due to CTA

**Figure 5:**
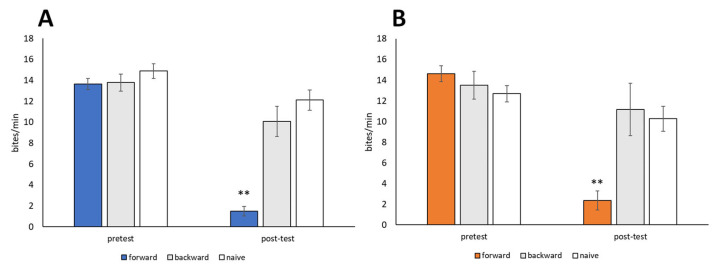
Memory formation of CTA in food-satiated Day −1 and food-deprived Day 1 snails. (**A**) Day −1 snails. Numbers of bites elicited by the 10 mM sucrose CS in the pretest and post-test sessions are shown. In the forward-conditioned group, the feeding response to the CS was significantly reduced during the CTA memory test session compared to the backward-trained and naive control snails (2-way ANOVA; N = 28, 14, and 9, respectively; *F*_(2,48)_ = 20.6, *p* < 0.01 for interaction; *F*_(2,48)_ = 34.7, *p* < 0.01 for conditioning; *F*_(1,48)_ = 66.77, *p* < 0.01 for test. Holm post hoc test, ** *p* < 0.01). (**B**) Day 1 snails. CTA memory was consolidated (2-way ANOVA; N = 19, 18, and 19, respectively; *F*_(2,53)_ = 21.58, *p* < 0.01 for interaction; *F*_(2,53)_ = 8.77, *p* < 0.01 for conditioning; *F*_(1,53)_ = 63.36, *p* < 0.01 for test. Holm post hoc test, ** *p* < 0.01).

Snails were trained using the 10-trial training (i.e., CS-US pairing) procedure described in Section 2.5 (CTA paradigm). The CS was a 10 mM sucrose solution, and the US was a 10 mM KCl solution. Backward-trained and naive control snails were also prepared. Both food-satiated snails (Day −1 snails) and food-deprived snails (Day 1 snails) were used. No significant difference in feeding responses to the CS was observed during the pretest session performed before training (*p* > 0.05, Figure 5). Following CTA training, the feeding responses elicited by the CS in the post-test session were significantly reduced in both Day −1 snails and Day 1 snails (forward in Figure 5, *p* < 0.01, N = 28 for Day −1 snails, N = 19 for Day 1 snails) compared to the pretest and post-test sessions of the backward-trained or naive control snails (N = 14 and 9 for Day −1 snails, N = 18 and 19 for Day 1 snails, Figure 5). Thus, *Lymnaea* learned CTA when the CS (sucrose) and US (KCl) were paired in a forward manner but not a backward manner.

### 3.5. Change in the Localization of LymFOXO Protein in Lymnaea CNS after Food Deprivation, CTA Training, and Insulin Application

**Figure 6:**
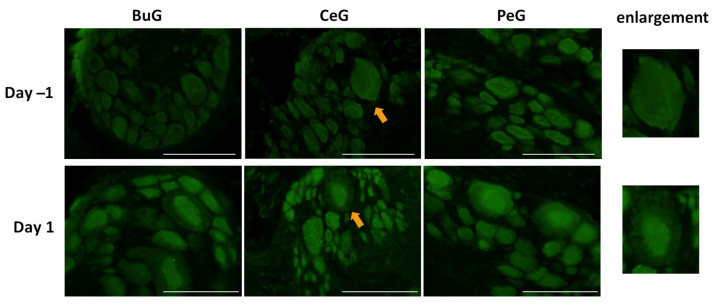
LymFOXO localization changes under food-deprivation conditions in CNS sections. BuG: buccal ganglion. CeG: cerebral ganglion. PeG: pedal ganglion. Green indicates the immunoreactivity of LymFOXO. Arrows indicate enlarged neurons (cerebral giant cells) shown in the right panels. Scale bars: 100 µm.

As shown in Figure 4, LymFOXO seemed to localize in the cytoplasm in food-satiated Day −1 snails. Thus, localization changes in LymFOXO under food-deprivation conditions were examined with immunohistochemistry using CNS sections of food-deprived Day 1 snails (Figure 6). Observations of the buccal ganglia (BuG), cerebral ganglia (CeG), and pedal ganglia (PeG), which are strongly involved in CTA [24], in Day −1 snails revealed that LymFOXO proteins were mainly localized in the cytoplasm: in five whole CNSs, LymFOXO was observed in the cytoplasm in three and in the nucleus in one. The localization was not clear in one CNS. On the other hand, strong localization in the nucleus was observed in Day 1 snails: in five whole CNSs, LymFOXO was observed in the nucleus in three and in the cytoplasm in one. The localization was not clear in one CNS. These results suggest that LymFOXO translocates into the nucleus under food-deprivation conditions.

**Figure 7:**
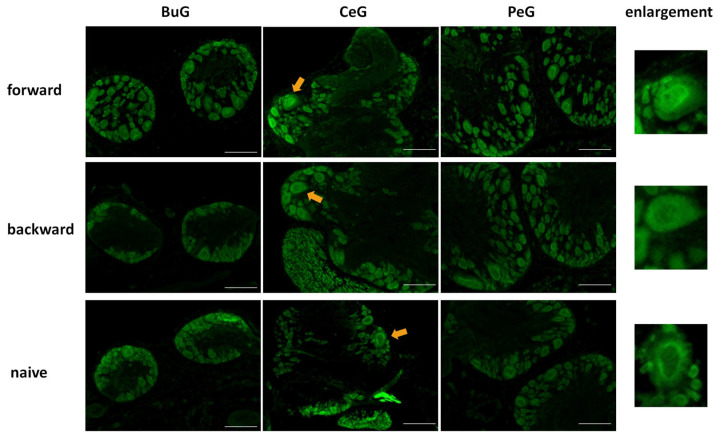
LymFOXO localization changes in the CNS sections of food-satiated Day −1 snails after CTA conditioning. BuG: buccal ganglion. CeG: cerebral ganglion. PeG: pedal ganglion. Green indicates LymFOXO immunoreactivity. Arrows indicate enlarged neurons shown in the right panels. The enlarged figure of a neuron from the CNS of a forward-conditioned snail shows that LymFOXO is present in the nucleus, whereas those from backward-trained and naive control snails show that LymFOXO is present in the cytoplasm. Scale bars: 100 µm.

Next, we examined changes in the localization of LymFOXO during CTA learning using immunohistochemistry (Figure 7). As mentioned above, LymFOXO was mainly present in the cytoplasm in Day −1 snails. After performing a CTA experiment on Day −1 snails, the CNSs were isolated and the localization of the LymFOXO proteins was examined for BuG, CeG, and PeG. In CTA forward-conditioned snails, LymFOXO tended to localize to the nucleus (N = 3), whereas it tended to localize to the cytoplasm in backward-trained (N = 2) and naive control (N = 2) snails. For example, the light green cells known to synthesize MIPs showed LymFOXO-immunopositive reactivity in their nuclei (arrows in Figure 7).

**Figure 8:**
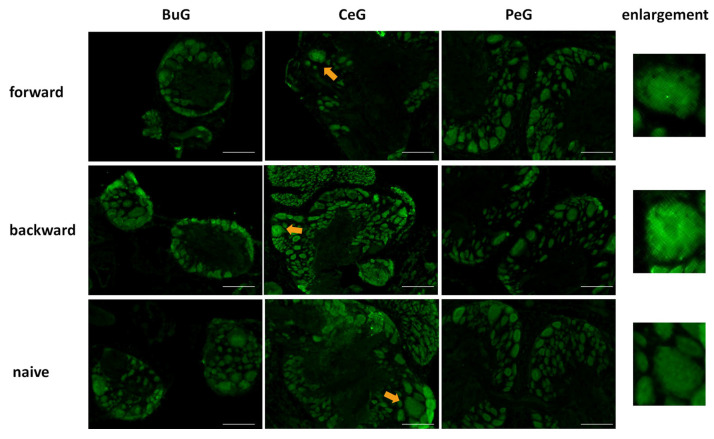
LymFOXO localization changes in CNS sections of food-deprived Day 1 snails after CTA conditioning. BuG: buccal ganglion. CeG: cerebral ganglion. PeG: pedal ganglion. Green indicates the immunoreactivity of LymFOXO. Arrows indicate enlarged neurons shown in the right panels. The enlarged figure of the CNS of a forward-conditioned snail shows that LymFOXO is present in the nucleus. Scale bars: 100 µm.

After the CTA learning experiments were performed on food-deprived Day 1 snails, the CNSs were isolated and the localization of LymFOXO proteins was examined in the BuG, CeG, and PeG (Figure 8). LymFOXO proteins were observed in the nucleus in the forward-conditioned CTA snails (N = 4) and mainly in the nucleus and often in the cytoplasm in the backward-trained (N = 3) and naive control (N = 3) snails.

**Figure 9:**
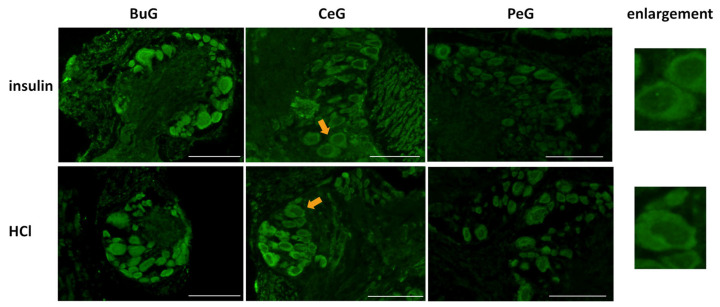
Localization changes of LymFOXO under insulin administration conditions in CNS sections of food-satiated Day −1 snails. BuG: buccal ganglion. CeG: cerebral ganglion. PeG: pedal ganglion. Green indicates the immunoreactivity of LymFOXO. Arrows indicate enlarged neurons shown in the right panels. Enlarged figures of both insulin and HCl administration show that LymFOXO is present in the cytoplasm. Scale bars: 100 µm.

Previous studies reported the involvement of insulin-like peptides in the memory consolidation of CTA in *Lymnaea* [20,21]. Therefore, we focused on food-satiated Day −1 snails, in which LymFOXO was present in the cytoplasm in the CNS neurons, and examined whether LymFOXO translocates into the nucleus following insulin administration (Figure 9). The CNSs were removed from Day −1 snails and immersed in 100 nM insulin or 120 nM HCl (vehicle control) for 1 h, followed by immunohistochemistry. Observations of the BuG, CeG, and PeG in the CNS sections revealed strong localization of LymFOXO proteins in the cytoplasm after soaking the CNSs in insulin (three of three CNSs). In the vehicle control, LymFOXO proteins localized in the cytoplasm as in the Day −1 samples without insulin application (three of three CNSs).

### 3.6. Change in the LymFOXO mRNA Expression Level in Lymnaea CNS after Food Deprivation and Insulin Application

**Figure 10:**
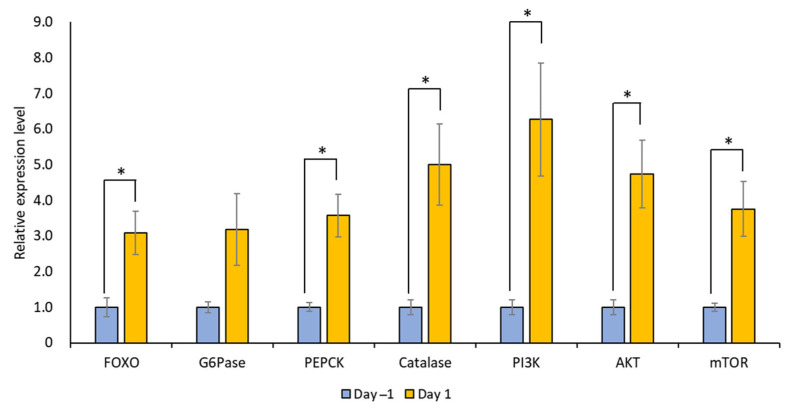
Changes in the mRNA expression levels of LymFOXO, LymG6Pase, LymPEPCK, LymCatalase, LymAKT, LymPI3K, and LymmTOR in the whole CNSs under different food deprivation conditions (i.e., food-satiated Day −1 snails and food-deprived Day 1 snails). LymG6Pase, LymPEPCK, and LymCatalase are target genes of LymFOXO. LymPI3K, LymAKT, and LymmTOR are insulin signaling pathway genes. The mRNA expression levels were normalized to β-actin and β-tubulin RNAs, and the expression of each gene is shown as the relative expression level (RQ), with that in Day −1 snails set as 1. * *p* < 0.05. N = 5 each.

The mRNA expression levels of LymFOXO, LymFOXO target genes (LymG6Pase, LymPEPCK, and LymCatalase), and insulin signaling pathway genes (LymPI3K, LymAKT, and LymmTOR) were measured for Day 1 and Day −1 snail CNSs using real-time PCR (N = 5 each). In general, G6Pase and PEPCK are involved in gluconeogenesis, and catalase is involved in oxidative stress [31]. PI3K, AKT, and mTOR are molecules downstream of the insulin cascade involved in the cell cycle, protein synthesis, and cell growth [32]. The levels of all these mRNAs, except LymG6Pase, increased significantly in food-deprived Day 1 snails compared to food-satiated Day −1 snails (Figure 10; *p* = 0.034 for LymFOXO, *p* = 0.126 for LymG6Pase, *p* = 0.017 for LymPEPCK, *p* = 0.033 for LymCatalase, *p* = 0.040 for LymPI3K, *p* = 0.023 for LymAKT, and *p* = 0.032 for LymmTOR). Thus, food deprivation upregulates LymFOXO mRNA, thereby promoting its target genes as a transcription factor, and upregulates the mRNAs of insulin signaling pathway genes. We should note, however, that the changes in the protein levels were not clear.

**Figure 11:**
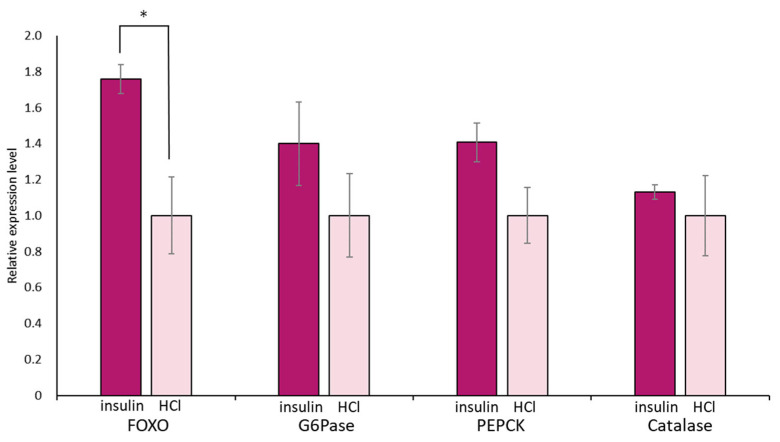
Changes in mRNA expression levels of LymFOXO, LymG6Pase, LymPEPCK, and LymCatalase in the whole CNSs after insulin administration to CNSs isolated from Day −1 snails. LymG6Pase, LymPEPCK, and LymCatalase are target genes of LymFOXO. The mRNA expression levels were obtained after normalizing to β-actin and β-tubulin RNAs, and the expression of each gene is shown as the relative expression level (RQ), with that of the control CNSs (application of HCl) set as 1. * *p* < 0.05. N = 4 each.

Again, we focused on food-satiated Day −1 snails, in which LymFOXO was present in the cytoplasm in the CNS neurons. The immunohistochemical data of LymFOXO proteins showed that no LymFOXO translocated from the neuronal cytoplasm to the nucleus (Figure 11). Thus, we examined whether LymFOXO mRNA and its target genes’ mRNAs were altered by insulin administration to the CNSs of Day −1 snails. The treatment of the CNSs (N = 4) with insulin significantly increased the mRNA expression level of LymFOXO compared to the CNSs (N = 4) treated with HCl (vehicle of insulin; *p* = 0.048 for LymFOXO, *p* = 0.332 for LymG6Pase, *p* = 0.118 for LymPEPCK, *p* = 0.650 for LymCatalase).

## 4. Discussion

The present results demonstrated that FOXO is present in *Lymnaea*. An analysis of the deduced amino acid sequence of LymFOXO showed that the AKT phosphorylation site reported in other species is conserved [17,33], so it is likely that the role of LymFOXO is similar between species. The translocation of FOXO into the nucleus under food deprivation conditions is also reported in other animals [31]. Because LymFOXO is localized in the nucleus during CTA learning, it may be involved in learning and memory mechanisms in *Lymnaea*. On the other hand, the insulin signaling cascade exists upstream of the FOXO responses, but even when insulin was administered to *Lymnaea* CNSs isolated from food-satiated snails, LymFOXO remained in the cytoplasm and did not translocate to the nucleus. These observations raise the possibility that both food deprivation and insulin are associated with enhanced learning performance in *Lymnaea* but that FOXO is involved in learning and memory independent of the insulin cascade. Our findings are consistent with previous findings that the activation of DAF-16 (*C. elegans* FOXO) independent of insulin/IGF-1 signaling is involved in learning in *C. elegans* [14,34].

Previous studies suggested that FOXO is degraded in the cytoplasm [35]. In the present study, insulin administration increased LymFOXO mRNA expression. Based on this observation, we predict that although insulin does not promote translocation from the cytoplasm to the nucleus in *Lymnaea*, it may upregulate LymFOXO gene expression, making it easier for FOXO to act when it enters the nucleus. Furthermore, it has been reported that the regulation of FOXO is complicated [36,37,38]. For example, although FOXO is modified through deacetylation, the functional consequences are less clear. The effect of deacetylation is the promotion of cellular survival under stress and protection against apoptosis by fine-tuning FOXO transcription. We need more work to understand the effect of deacetylation on FOXO’s function.

In addition to FOXO, another important transcription factor, CREB, exists downstream of the insulin cascade [39]. In the *C. elegans* nervous system, CREB is reported to be upregulated as a target gene for daf-16 (i.e., FOXO) [11]. On the other hand, it has been reported that FOXO’s function is regulated via its acetylation by a CREB co-activator, CBP [40,41], and that FOXO gene expression is regulated by a CREB co-activator, P300, during fasting [42]. In *Lymnaea stagnalis*, LymCREB and its related factors have been reported [43,44]. The cDNA sequences of LymCREB1 and LymCREB2 were identified and correspond to transcriptional activators (e.g., mammalian CREB1 and *Aplysia* CREB1a) and transcriptional repressors (e.g., human CREB2, mouse activating transcription factor-4, and *Aplysia* CREB2), respectively. In situ hybridization of the CNS revealed that relatively few neurons showed strong positive signals for LymCREB1, whereas all the neurons contained LymCREB2. Taken together with the present results, FOXO signaling may function in all neurons, whereas CREB signaling may play an important role in some specific neurons in the *Lymnaea* CNS.

Although the real-time PCR data for each ganglion showed no significant differences in the expression level of LymFOXO mRNA, the expression level of this mRNA tends to be higher in ganglia that contain many endocrine cells, such as the CeG, PaG, and VG. Furthermore, our present immunohistochemistry data suggest the following: (1) Transcription factors related to long-term memory such as FOXO and CCAAT-enhancer binding protein (C/EBP) are expressed in the BuG motor neurons, suggesting that they influence the regulation of feeding responses after CTA learning is established. (2) In the CGCs, CREB is activated by protein kinase A and conjugates with CBP to increase its transcriptional activity [43,44]. On the other hand, FOXO is inhibited by protein kinase A and the acetylation of CBP [45,46,47,48]. Thus, in the CGCs, CREB and FOXO may have opposing effects. In *Lymnaea*, the fact that the light green cells known to synthesize MIPs contain LymFOXO and the fact that LymFOXO is located downstream of the MIP cascade suggest that the relationship between MIPs and LymFOXO may be meaningful.

Furthermore, hyperactivation of the insulin/IGF-1 signaling pathway through mutations in PTEN/DAF-18 enhances the learning ability compared to wild-type worms. Mutants lacking FOXO/DAF-16, however, exhibit impaired learning. The learning-enhancing effects of insulin receptor/DAF-2 act via the production of phosphatidylinositol 3,4,5-trisphosphate (PIP3) rather than via the function of the FOXO/DAF-16 transcription factor. These findings indicate that the signaling pathway from DAF-2 that affects learning diverges between PIP3 production and DAF-16 function, suggesting that DAF-16 activity, which is independent of insulin/IGF-1 signaling, is involved in learning [14].

FOXO is involved in neuroplasticity in *C. elegans*, *Drosophila*, and mice and is involved in autophagy in mice [49]. In *Lymnaea*, neuroplasticity between the CGCs and the following neurons can be observed after insulin application [21]. An autophagy mechanism has also been observed in serotonin (5-HT) signaling, where the amount of tryptophan, a precursor of 5-HT, is increased by degrading proteins in the CNS [25]. Thus, LymFOXO has several functions that have been reported in other species.

Recently, an interesting paper was published on *C. elegans* [50]. When parental (P0) animals were trained in aversive associative learning, the memory was transmitted to F1 and F2 offspring. To corroborate this, the odor-triggered cellular stress response was also inherited, as indicated by the translocation of FOXO to the nucleus.

## 5. Conclusions

LymFOXO was localized in the cytoplasm of CNS neurons in food-satiated snails, whereas it was mainly observed in the nucleus in food-deprived snails. When CTA learning was acquired, LymFOXO was also translocated to the nucleus, even in food-satiated snails, whereas it remained in the nucleus in food-deprived snails. Insulin administration did not cause the translocation of LymFOXO into the nucleus. Food deprivation prepares FOXO to work in the nucleus, and snails learn better if they learn CTA in this condition. Insulin application did not directly affect the location of LymFOXO proteins, suggesting that insulin stimulates pathways other than the molecules downstream of LymFOXO.

## Figures and Tables

**Table 1:** Primers used to determine sequences.

Gene Name	Accession Number	Forward or Reverse	Primer Sequence 5′-3′
FOXO (i)	FX227609.1	Forward	CGAACCCCAAACAAGAGCG
	FX199776.1	Reverse	TGCTGCTTTGGGTATGGAGG
FOXO (ii)	FX199776.1	Forward	GGAGGTGACCAATTCCAGCA
	FX217506.1	Reverse	CCCAGAGGTGAGTGGTTTCC
3′ RACE adaptor	-	-	GCGAGCACAGAATTAATACGACTCATATAGGTTTTTTTTTTTTVN
3′ RACE FOXO-specific outer	-	Forward	TCTGTGAACACACAGCAGCA
3′ RACE outer	-	Reverse	GCGAGCACAGAATTAATACGACT
3′ RACE FOXO-specific inner	FX217506.1	Forward	GCAGCAGGCTTCTATACTTCG
3′ RACE inner	-	Reverse	CGCGGATCCGAATTAATACGACTCACTATAGG

**Table 2:** List of FOXO-like proteins and their accession numbers for the neighbor-joining tree.

Gene Name	Accession Number
*Aplysia californica* forkhead box protein O	XP_005112460.1
*Biomphalaria glabrata* forkhead box protein O-like	KAI8758266.1
*Bombyx mori* forkhead box sub-group O	AFD99125.1
*Caenorhabditis elegans* DAF-16	AAC47803.1
*Crassostrea hongkongensis* forkhead box O protein	APG29285.1
*Drosophila melanogaster* forkhead box, sub-group O, isoform B	NP_996205.1
*Elysia marginata* forkhead box O protein	GFR80317.1
*Gigantopelta aegis* forkhead box protein O-like	XP_041351714.1
*Haliotis discus hannai* FoxO	QOI08485.1
*Homo sapiens* forkhead box protein O1	NP_002006.2
*Homo sapiens* forkhead box protein O3 isoform 1	NP_001446.1
*Homo sapiens* forkhead box protein O4 isoform 1	NP_005929.2
*Homo sapiens* forkhead box protein O6	NP_001278210.2
*Lymnaea stagnalis* FOXO	LC773945.1
*Mus musculus* forkhead box protein O1	NP_062713.2
*Mus musculus* forkhead box protein O3	NP_001363896.1
*Mus musculus* forkhead box protein O4	NP_061259.1
*Mus musculus* forkhead box protein O6	NP_918949.1
*Paramuricea clavata* forkhead box O-like isoform X1	CAB3987597.1
*Pomacea canaliculata* forkhead box protein O-like	XP_025097678.1
*Sinonovacula constricta* FOXO	AYW35875.1
*Xenopus laevis* forkhead box protein O1	NP_001086417.1
*Xenopus laevis* forkhead box protein O3	NP_001086418.1
*Xenopus laevis* forkhead box protein O4 L homolog	NP_001154870.1
*Xenopus laevis* forkhead box protein O6 S homolog	NP_001152754.1

**Table 3:** Accession numbers of query sequences, accession numbers of *Lymnaea* molecules, and amino acid identities of insulin-cascade genes.

Gene Name	Accession Number of Query Sequence	Accession Number of *Lymnaea*	Amino Acid Identity
LymG6Pase	*Aplysia californica* G6Pase (XM_005094411.3)	FX188931.1	60.0%
LymPEPCK	*Crassostrea gigas* PEPCK (NM_001305293.1)	FX183226.1	73.7%
LymCatalase	*Aplysia californica* Catalase (XM_005090003.3)	FX188459.1	79.1%
LymPI3K	*Aplysia californica* PI3KCA (XM_013086940.2)	FX182614.1	84.3%
LymAKT	*Aplysia californica* AKT (XM_035970173.1)	FX187320.1	94.3%
LymmTOR	*Aplysia californica* mTOR (XM_013080183.2)	FX180237.1	90.2%

**Table 4:** Primers used for real-time PCR experiments.

Gene Name	Accession Number	Forward or Reverse	Primer Sequence 5′-3′	Product Size (bp)
LymFOXO	LC773945.1	Forward	CCTCCATACCCAAAGCAGCA	182
		Reverse	CACTGAACAAGCCCTTTGCC	
LymG6Pase	FX188931.1	Forward	CCACGTCAACGGTGAACCTA	258
		Reverse	AGCACACGTTTTCCGACTGA	
LymPEPCK	FX183226.1	Forward	GATGCCCACGACCAGTACAA	192
		Reverse	TCCATCCCTCATCTCTGGCA	
LymCatalase	FX188459.1	Forward	TGGGACTTCTTCACTCTGCG	136
		Reverse	TCTCAGCCTTGTTGACCAGC	
LymPI3KCA	FX182614.1	Forward	CTCAAGTCCACGATGGGACC	153
		Reverse	AACAGCTGAAGATTGCCCCA	
LymAKT	FX187320.1	Forward	GATGACCAGCAGACTGGACC	119
		Reverse	CTGCCGATGTGCTGAGGTAA	
LymmTOR	FX180237.1	Forward	TGCAGCAGATCCAGCAGAAA	274
		Reverse	AAGCGGTTATCTCGCCTAGC	
18s rRNA	Z73984.1	Forward	CTCCTTCGTGCTAGGGATTG	106
		Reverse	GTACAAAGGGCAGGGACGTA	
β-actin	KX387884.1	Forward	GCAGAAGGAAATCACAGCACTGG	114
		Reverse	GTGGAGAGAGAGGCAAGGATGG	
β-tubulin	KX387888.1	Forward	CAAGCGCATCTCTGAGCAGTT	108
		Reverse	TTGGATTCCGCCTCTGTGAA	

## Data Availability

All data that support the findings of this study are available from the corresponding authors upon reasonable request.
